# An atypical presentation of immune checkpoint inhibitor associated myositis with normal creatine kinase: a case report

**DOI:** 10.3389/fimmu.2025.1661534

**Published:** 2025-10-08

**Authors:** Monika A. Satoskar, Anthony G. Mansour, Richard Wu, Yuanquan Yang, Mary H. Caldwell, Alexa S. Meara

**Affiliations:** ^1^ Department of Internal Medicine, College of Medicine, The Ohio State University, Columbus, OH, United States; ^2^ Department of Internal Medicine Division of Medical Oncology, Department of Internal Medicine, James Comprehensive Cancer Center, The Ohio State University, Columbus, OH, United States; ^3^ Department of Internal Medicine Division of Rheumatology and Immunology, The Ohio State University, Columbus, OH, United States

**Keywords:** immunotherapy, immune checkpoint inhibitor, myositis, immune-related adverse event, aldolase, creatine kinase (CK)

## Abstract

Immune checkpoint inhibitors (ICIs) have been revolutionary in the field of cancer therapeutics. Myositis is a known rheumatic immunotherapy related adverse event with a fatality rate of 26.8% when associated with myasthenia gravis and 51.3% when associated with myocarditis. Typically, creatine kinase (CK) is elevated in ICI-myositis, thus normal CK levels in such cases may delay diagnosis and treatments in such patients. We report 2 cases of patients diagnosed with ICI-myositis after ipilimumab/nivolumab treatment for metastatic renal cell carcinoma and metastatic melanoma. Their lab studies showed normal CK values but elevated aldolase, which led to the ICI-myositis diagnosis and steroid treatment. Early recognition and treatment with steroids led to symptom improvement. These 2 cases highlight an atypical presentation of ICI-myositis with normal CK but elevated aldolase levels, suggesting aldolase may be a sensitive parameter in diagnosing ICI-myositis.

## 1 Introduction

Immune checkpoint inhibitors (ICIs) have been revolutionary in cancer therapeutics. They block certain immune regulatory checkpoint proteins, allowing for the body’s immune system to target and attack the cancer cells. However, these powerful agents are also known to cause various adverse effects on organs, known as immune-related adverse events (irAEs). Myositis is a known rheumatic irAE. Although relatively rare, accounting for approximately 0.38-0.6% of irAEs, it is one of the most severe forms of rheumatic irAEs with a case fatality rate of 26.8% when associated with myasthenia gravis and 51.3% when associated with myocarditis (1). Studies have shown a significantly increased risk of developing myositis in patients treated with ICIs compared to those not treated with ICIs (2).

There is limited data on ICI-associated myositis in terms of clinical presentation and laboratory parameters required to establish a diagnosis. Typically, in ICI-myositis, creatine kinase (CK) is elevated (2). Here, we present 2 cases of ICI-myositis with a normal CK and elevated aldolase levels, both of which responded well to steroids.

## 2 Case presentations

### 2.1 Case 1

We report a case of a 72-year-old male with a history of paroxysmal atrial fibrillation who presented with cough and dyspnea for 2 months. His computed tomography (CT) chest scan revealed multiple solid noncalcified bilateral pulmonary nodules, and an indeterminate 6.5-cm left renal mass with multiple heterogenous left perinephric nodules concerning for regional spread of disease and associated left urothelial thickening. A lung biopsy revealed metastatic renal cell carcinoma, clear cell type.

**Figure 1:**
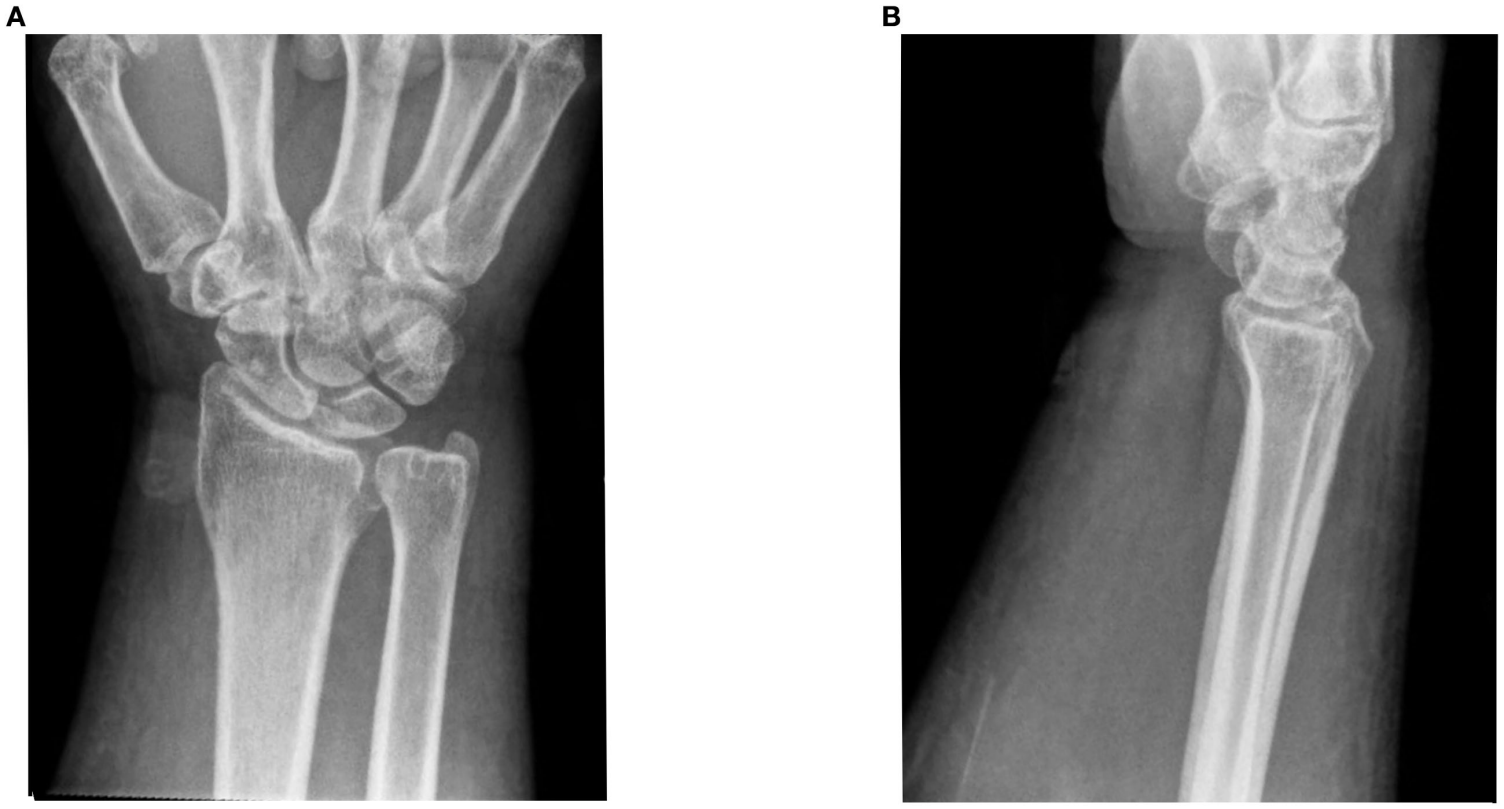
Right wrist x-rays. **(A)** DA and lateral filme of right wrist ob Im **(B)** Lateral film diffuse soft tissue inflammation.

**Figure 2:**
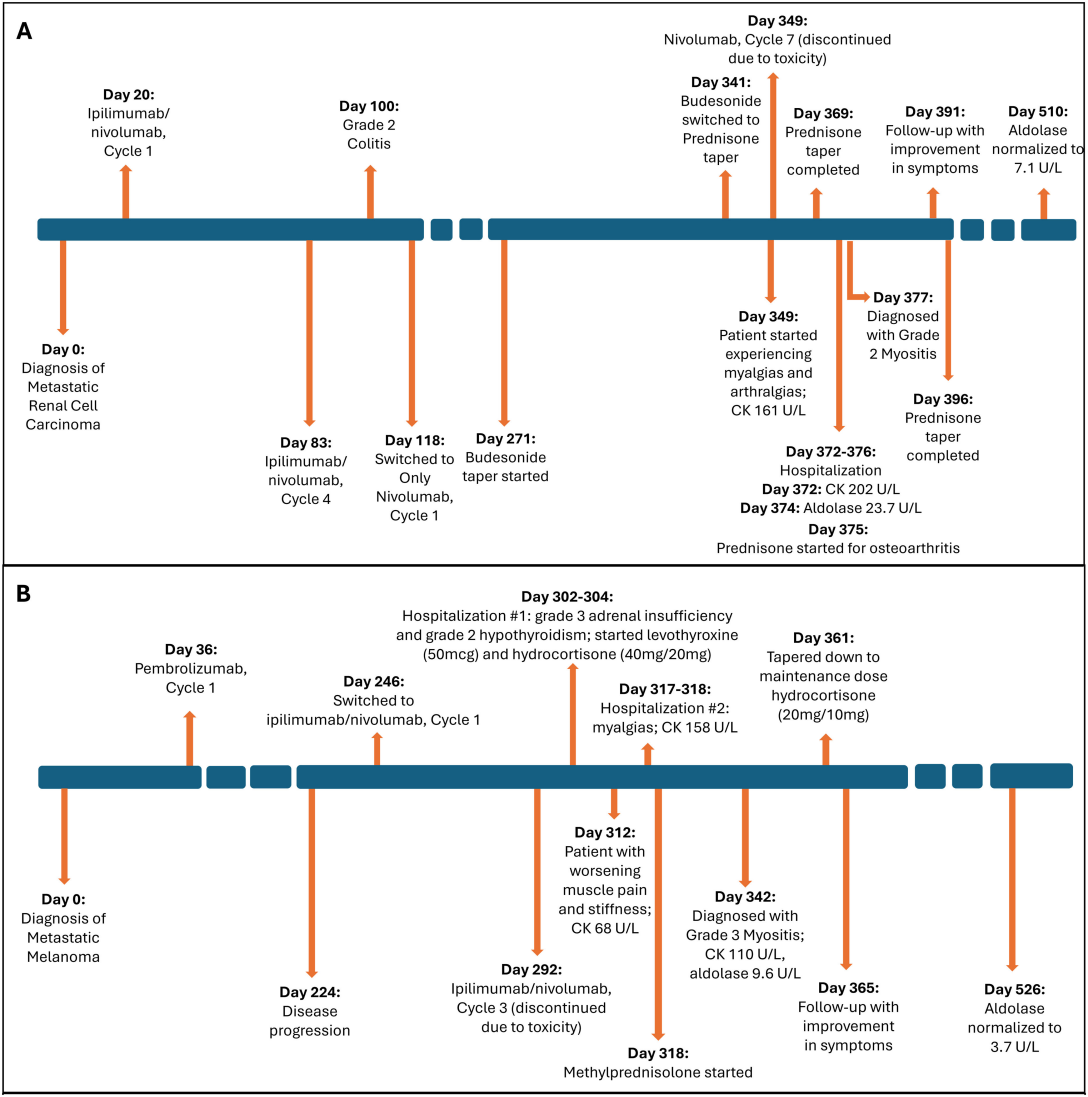
Timeline of event. **(A)** Case 1. **(B)** Case 2. Figure illustrating timeline of major events including diagnosis, treatment, laboratory parameter for the 2 cases. CK, creatine kinase.

He was started on ipilimumab (91mg)/nivolumab (274mg). He completed 4 cycles (9 weeks) with partial response. He developed grade 2 colitis (per Common Terminology Criteria for Adverse Events [CTCAE], version 5) (3) after the 4 treatment cycles and was switched to nivolumab alone (480 mg). He was treated with a prolonged budesonide taper (entailing 9 mg daily for 2 weeks, then 6 mg daily for 2 weeks, then alternation between 6 mg and 3 mg daily for 2 weeks, then 3 mg daily for 2 weeks, then alternation between 3 mg and 0 mg daily for 2 weeks). This was followed by a prednisone taper (entailing 5 mg daily for 2 weeks, then 2.5 mg daily for 2 weeks). He continued immunotherapy until cycle 7 (33 weeks) after which he developed symptoms of diffuse myalgias, joint stiffness, and fatigue. The CK level obtained at that time was normal (161 U/L). He became unable to perform his activities of daily living requiring hospital admission within that month. This was approximately 5 days after completing the prednisone taper and 8 months after starting nivolumab alone. His labs were notable for white blood cell count (WBC) (15.9 K/uL), C-reactive protein (26 mg/L), elevated transaminases (aspartate aminotransferase [AST], 99 U/L and alanine aminotransferase [ALT], 102 U/L), and normal CK (202 U/L), all of which were collected around 101 days from when budesonide was started. The autoimmune workup including rheumatoid arthritis (RA), and systemic lupus erythematosus (SLE) was negative (**Table 1**). Viral causes tested included hepatitis C which was negative. A right wrist x-ray was obtained for further evaluation of severe wrist pain and stiffness which showed diffuse soft tissue swelling (**Figure 1**). Initially, symptoms were thought to be due to osteoarthritis. He was discharged with prednisone 20mg daily for 7 days, which was tapered to 5mg daily over 3 weeks. The aldolase level (which was collected approximately 103 days from when the budesonide course was started) was found to be elevated at 23.7 U/L (reference range: < 7.7 U/L). He was diagnosed with grade 2 myositis (**Figure 2**) (3). Grade was determined according to the CTCAE v.5 which was based on severity of symptoms and interference with activities of daily living (3). Muscle MRIs and electromyograms (EMG) were not performed. A muscle biopsy was not completed since the patient already received steroid treatment with budesonide. Given improvement in symptoms, he was continued on the steroid regimen and tolerated it well. At his 2-week follow-up visit, his myalgia had improved. His aldolase level gradually declined back to normal (7.1 U/L) over the following 5 months. Immunotherapy was discontinued given 2 irAEs. Staging scans that were obtained after discontinuing immunotherapy revealed partial response. The patient remains off treatment and on surveillance.

**Table 1:** Summary of clinical and laboratory parameters.

Clinical/Lab Parameter	Case 1	Case 2
Cancer type	Metastatic renal cell carcinoma, clear type	Metastatic melanoma (SOX10 positive, Melan-A positive, S100 protein positive)
Immune Checkpoint Inhibitor (ICI) therapy	Ipilimumab (91 mg)/nivolumab (274 mg), switched to nivolumab (480 mg) alone	Pembrolizumab (400 mg), switched to ipilimumab (225 mg)/nivolumab (72.9 mg)
Treatment for Myositis	Prednisone 20 mg daily, tapered to 5 mg daily	Methylprednisolone (1 mg/kg) 80 mg daily, tapered to maintenance hydrocortisone (20 mg in morning/10 mg in evening)
Creatine kinase (U/L) at diagnosis (ref: 30-220)	202	110
Aldolase (U/L) at diagnosis (ref: <7.7)	23.7	9.6
Autoantibodies		
Rheumatoid factor (IU/mL) (ref: <10)	<10	<10
Cyclic citrullinated peptide (U/mL) (ref: <0.5)	<0.5	<0.5
Antinuclear antibody	Negative	Negative
Anti-myeloperoxidase antibody	Not Obtained	Negative
Anti-proteinase 3 antibody	Not Obtained	Negative
Anti-neutrophil cytoplasmic antibody	Not Obtained	Negative
Inflammatory markers		
Erythrocyte sedimentation rate (mm/hr) (ref: <20)	6	2
C-reactive protein (mg/L) (ref: <10)	26.08	0.64
Liver functions tests		
AST (U/L) (ref: 10-39)	99	49
ALT (U/L) (ref: 10-52)	102	91
White blood cell count (K/uL) (ref: 3.73-10.10)	15.87	10.52

### 2.2 Case 2

**Figure 3:**
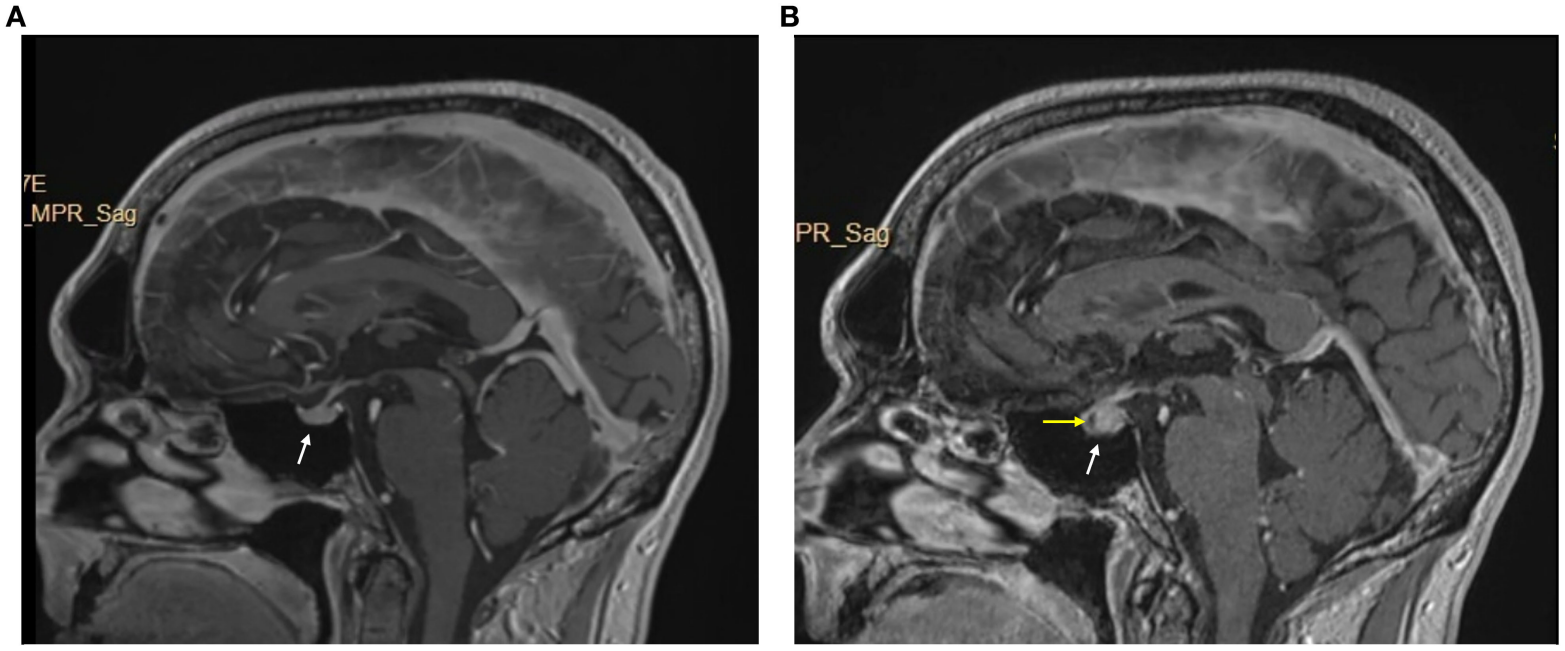
Brain MRI w/wo contrast pre- and post-immunotheraphy. **(A)** pre-immunotheraphy initiation; **(B)** post-immunotheraphy initiation.

We report a case of a 60-year-old male with no significant medical history who presented with left axillary mass with pain for about 1 week. A positron emission tomography-CT (PET-CT) scan revealed hypermetabolic activity in the left axillary mass. A biopsy revealed metastatic stage IV melanoma of unknown origin (SOX10 positive, Melan-A positive, S100 protein positive) with a BRAF p.V600E mutation. A brain magnetic resonance imaging (MRI) scan was completed for staging, which revealed no brain metastases (**Figure 3**).

He began pembrolizumab (400 mg) as neoadjuvant therapy and completed 5 cycles (24 weeks), with suboptimal response. The treatment was changed to ipilimumab (225 mg)/nivolumab (72.9 mg) as there was disease progression with metastasis to lymph nodes with extracapsular extension. After completing 3 cycles (7 weeks), he developed symptoms of diffuse muscle pain and fatigue requiring hospitalization. He was found to have abnormal blood chemistries including hyponatremia (sodium [Na], 118 mmol/L), hypocortisolism (cortisol, 1.01 mcg/dL), and hypothyroidism (thyroid-stimulating hormone [TSH], 0.274 uIU/mL, free thyroxine [T4], 0.66 ng/dL, free triiodothyronine [T3], 2.2 pg/mL). A brain MRI revealed an enlarged pituitary gland compared to his prior scan (from 9 months earlier), and with a new hypo-enhancing pituitary lesion (**Figure 3**). These findings were consistent with hypophysitis resulting in grade 3 adrenal insufficiency and grade 2 hypothyroidism (3). At this time, his myalgias were attributed to this endocrinopathy, and he was started on hydrocortisone (40 mg in morning/20 mg in evening) which was tapered to a maintenance dose (20 mg in morning/10 mg in evening) and levothyroxine (50 mcg).

At his 1-week follow-up visit, while on the maintenance dose, his muscle pain and stiffness worsened. Unsure if his endocrinopathy was worsening or if he was not receiving the correct treatment, further blood work was completed. He had a normal CK level (68 U/L), so myositis was ruled out. Despite myositis being ruled out, he was hospitalized again for persistent symptoms. This was approximately 10 weeks after starting ipilimumab/nivolumab. At that time, his labs were notable for a normal CK (158 U/L) and elevated transaminases (AST, 49 U/L and ALT, 91 U/L). His autoimmune workup including RA, SLE was negative otherwise (**Table 1**). Hepatitis C was negative. He was ultimately discharged with methylprednisolone (1mg/kg) for debilitating pain attributed to an unknown autoimmune/inflammatory syndrome from immunotherapy treatment. This was tapered over the next 6 weeks back to his prior maintenance dose. During his admission, a muscle biopsy was not completed as he had already received steroid treatment for his hypophysitis. Additionally, muscle MRIs and EMGs were not performed. He was seen at a follow-up visit 3 weeks after discharge. His aldolase level was checked (which was approximately 24 days from when methylprednisolone was started) and was elevated (9.6 U/L). His CK level remained normal (110 U/L). He was diagnosed with grade 3 myositis (**Figure 2**) (3). He continued the methylprednisolone taper since it was well-tolerated and resumed his maintenance hydrocortisone dose around 3 weeks later. At follow-up visit that week, he had improvement in myalgias. His aldolase level normalized to 3.7 U/L over the following 6 months. Immunotherapy was discontinued given 2 irAEs requiring hospitalization. He remains on surveillance with no evidence of active disease on staging scans.

## 3 Discussion

These 2 cases highlight an atypical presentation of immunotherapy-related myositis with normal CK levels but elevated aldolase levels that responded to steroid treatment.

ICI-myositis typically occurs with CK elevation, which was not the case in this report; it is important to note that the aldolase level was elevated, suggesting that this may be a sensitive laboratory marker in the diagnosis of ICI-myositis.

Aldolase elevation suggests damage of early regenerating muscle cells, which is not well understood; however, there are a few descriptive cases in rheumatology (4). In a study, aldolase A mRNA that was isolated from cultured human myoblasts and protein expression was highest in early differentiating myoblasts and remained elevated throughout differentiation, in contrast to CK mRNA and protein levels which remained low initially but increased as differentiation progressed (4). There have been several clinical studies showing a similar pattern of normal CK and elevated aldolase levels. In one study of 66 patients with dermatomyositis, 17 (26%) of them had normal CK with elevated aldolase (5). In another study, there were 12 patients with polymyositis confirmed on muscle biopsy, all of whom had a normal CK level with an elevated aldolase similar to our cases (6). There was another study of 34 patients with various myopathies including dermatomyositis, overlap myositis, and nonspecific myopathy. All of these patients were also found to have normal CK with elevated aldolase levels (7). This suggests that an elevated aldolase is a sensitive marker for many of these autoimmune myopathies and potentially ICI-myositis.

There have been a few studies exploring aldolase levels in patients with myopathy related to ICI use. In one retrospective study with 36 patients who either had ICI-myositis alone or with overlap manifestations including myocarditis and myasthenia gravis, 94% of the cohort had elevated CK levels and 26/27 patients tested had an elevated aldolase level (8). Two patients had a normal CK level, one of whom had elevated aldolase similar to our cases (8). Another retrospective study comparing patients with ICI myopathy to those with immune-mediated necrotizing myopathy (not exposed to immunotherapy) showed modest elevation in both aldolase and CK levels among the ICI myopathy group, but the elevation was far more prominent in the immune-mediated necrotizing myopathy group (9). Similar to our cases, 7/22 patients in the ICI myopathy group had normal CK levels, of which 2 had elevated aldolase levels (9). In contrast to our case series findings, there was another case series, of 2 patients who developed ICI-myositis following treatment with pembrolizumab who had both elevated CK and aldolase levels (10).

The variation in presentation of CK and aldolase levels seen in the literature further emphasizes the need for additional studies to better understand the diagnostic pattern for ICI-myositis. There is limited evidence on the diagnostic criteria for ICI-myositis. Presentation of ICI-myositis with a normal CK and elevated aldolase levels is rare. It may be that myositis is missed due to the diagnosis being dependent on an elevated CK level, which can lead to a delay in the diagnosis and treatment of such patients. This raises the question of whether aldolase should become part of the initial testing of patients for whom ICI-myositis is suspected.

The exact mechanism for an isolated elevated aldolase is unclear. A possible reason for this may include the extent of muscle fiber splitting and vacuolization of muscle fibers. Studies have shown patients with elevated CK have more frequent fibrillation potentials on EMG which correlate to presence of necrotic fibers, fiber splitting or vacuolization (7). Another explanation may be whether there is involvement of the perimysium. A study showed that 11/12 patients (92%) with isolated aldolase elevation had perimysial pathology (11). There needs to be further investigations exploring this to better understand the underlying mechanisms.

It is also important to note that both of our cases had prior steroid exposure for another ICI-related event, and so it is unclear whether the CK level was elevated before and normalized immediately upon starting steroids, or whether it was normal to begin with. Conversely, aldolase not only was elevated, but remained elevated for several months after the completion of steroids.

Studies have shown that patients in the acute setting of glucocorticoid-induced myopathy can have high aldolase levels with improvement within 3-4 weeks of discontinuing or reducing the steroid dose (12–14). This is typically seen at doses exceeding 60mg prednisolone daily (or equivalent) (12–14). Chronic steroid myopathy, however, is typically seen with steroid use for more than 4 weeks and in doses exceeding 10mg prednisolone daily (or equivalent) (12–14). In the setting of chronic steroid myopathy, aldolase levels have been shown to be typically normal (13). In the setting of inflammatory myopathies, or in our case of ICI-myositis, and prior steroid exposure, it becomes challenging to distinguish whether the aldolase elevation is due to the underlying disease or muscle damage related to the steroid. Studies have shown that EMG or muscle biopsy findings can aid in distinguishing between the two pathologies, however, these additional tests were not completed among our patients (13). Therefore, our study findings suggest that aldolase elevation may be a sensitive marker for ICI-myositis in the setting of prior steroid exposure. It is important to note, however, that for Case 1, he was started on steroids (with budesonide) over 10 weeks in advance from when aldolase was measured and tapered (**Figure 2**, box A). In this setting of chronic steroid use, we would expect to see normal aldolase levels. Additionally, after the completion of the steroid course (Case 1) or taper down to the maintenance dose (for adrenal insufficiency in Case 2), the aldolase level remained elevated for over 20 weeks which is atypical for steroid-induced myopathy, although further tests such as EMGs and muscle biopsies are needed to confirm.

ICI-myositis typically presents with progressive upper and lower extremity weakness within 2-3 months of ICI initiation, along with myalgias and fatigue. However, there can be additional clinical manifestations including ocular, cardiac, and even neurologic involvement. Studies report that oculo-bulbar symptoms can present in up to 25% of patients with ICI-myositis, an uncommon presentation seen with idiopathic inflammatory myopathies, unrelated to ICI use (15). These symptoms present as ptosis, ophthalmoplegia, dysphagia, and potentially dyspnea from diaphragm involvement. Additionally, studies report that myocarditis can concurrently occur in patients with ICI-myositis (2, 16, 17). In a study of 9,088 patients, around 36 (0.40%) patients had ICI-myositis, among which 14 (39%) patients had concurrent myocarditis (8). Neither of our two patients developed myocarditis and all cardiac work-up including EKG, troponins, and echocardiograms have been normal. Given these broad clinical manifestations and organ involvement that can be fatal if left untreated, it is even more important to recognize ICI-myositis so that appropriate therapy can be initiated.

The strengths of this case series include the fact that both patients at baseline had relatively few co-morbidities and took few medications. Medications they took were not commonly associated with myositis, thus limiting the confounding bias. One of the limitations of this case series is the lack of muscle biopsies to confirm the diagnosis of myositis; our diagnosis was based on clinical presentation and elevated aldolase levels. The inflammation seen on right wrist imaging for Case 1, however, did support the diagnosis. Additionally, both patients had prior steroid exposure for an irAE other than myopathy. This may have contributed to the normalization of CK levels at the time of ICI-myositis diagnosis and may also potentially confound the aldolase elevation seen. Further testing including EMG, muscle biopsies are needed to confirm.

## 4 Patient perspective

Both patients at follow up 2-3 weeks after diagnosis of ICI-myositis, and on steroid treatment, noted significant improvement in myalgias and ability to perform daily activities of living.

## 5 Conclusion

Myositis is a rare but potentially severe irAE. Early identification of the clinical symptoms and understanding the varied clinical manifestations is key in recognizing ICI-myositis and initiating appropriate treatment. This case series highlights that, along with clinical features, laboratory abnormalities, including aldolase elevation despite normal CK levels, may further aid in the early identification of ICI-myositis, especially in the setting of prior steroid exposure.

## Data Availability

The original contributions presented in the study are included in the article/supplementary material. Further inquiries can be directed to the corresponding author.
